# iTRAQ-Based Proteomic Analysis of Ogura-CMS Cabbage and Its Maintainer Line

**DOI:** 10.3390/ijms19103180

**Published:** 2018-10-15

**Authors:** Fengqing Han, Xiaoli Zhang, Limei Yang, Mu Zhuang, Yangyong Zhang, Zhansheng Li, Zhiyuan Fang, Honghao Lv

**Affiliations:** 1 Institute of Vegetables and Flowers, Chinese Academy of Agricultural Sciences, Key Laboratory of Biology and Genetic Improvement of Horticultural Crops, Ministry of Agriculture, Beijing 100081, China; feng857142@163.com (F.H.); yanglimei@163.com (L.Y.); zhuangmu@163.com (M.Z.); zhangyangyong@163.com (Y.Z.); lizhansheng@163.com (Z.L.); fangzhiyuan@163.com (Z.F.); 2 Tianjin Kernel Vegetable Research Institute, The National Key Laboratory of Vegetable Germplasm Innovation, The Enterprise key Laboratory of Tianjin Vegetable Genetics and Breeding, Jinjing Road, Xiqing District, Tianjin 300384, China; zxl19871009@163.com

**Keywords:** *Brassica oleracea*, Ogura-CMS, iTRAQ, transcriptome, pollen development

## Abstract

Ogura cytoplasmic male sterility (CMS) contributes considerably to hybrid seed production in *Brassica* crops. To detect the key protein species and pathways involved in Ogura-CMS, we analysed the proteome of the cabbage Ogura-CMS line CMS01-20 and its corresponding maintainer line F01-20 using the isobaric tags for the relative and absolute quantitation (iTRAQ) approach. In total, 162 differential abundance protein species (DAPs) were identified between the two lines, of which 92 were down-accumulated and 70 were up-accumulated in CMS01-20. For energy metabolism in the mitochondrion, eight DAPs involved in oxidative phosphorylation were down-accumulated in CMS01-20, whereas in the tricarboxylic acid (TCA) cycle, five DAPs were up-accumulated, which may compensate for the decreased respiration capacity and may be associated with the elevated O_2_ consumption rate in Ogura-CMS plants. Other key protein species and pathways involved in pollen wall assembly and programmed cell death (PCD) were also identified as being male-sterility related. Transcriptome profiling revealed 3247 differentially expressed genes between the CMS line and the fertile line. In a conjoint analysis of the proteome and transcriptome data, 30 and 9 protein species/genes showed the same and opposite accumulation patterns, respectively. Nine noteworthy genes involved in sporopollenin synthesis, callose wall degeneration, and oxidative phosphorylation were presumably associated with the processes leading to male sterility, and their expression levels were validated by qRT-PCR analysis. This study will improve our understanding of the protein species involved in pollen development and the molecular mechanisms underlying Ogura-CMS.

## 1. Introduction

Cabbage (*Brassica oleracea* L. var. *capitata*) is an important leafy vegetable cultivated worldwide, and it provides substantial amounts of fibre, vitamins, mineral elements and health-promoting nutrients. The Food and Agriculture Organization of the United Nations reported that the global harvested area of vegetables in 2014 was 20,119,000 ha, with cabbage and other cole crops accounting for approximately 12% of the total (2,470,000 ha; see http://faostat3.fao.org/).

Commercially available cabbage mainly consists of hybrid cultivars because of their significant levels of heterosis. Cross-pollination in hybrid seed production is mainly accomplished using male sterility and self-incompatibility. Self-incompatibility has several limitations, such as poor seed purity and high costs of parental reproduction, whereas male sterility is generally more reliable and economically effective [1,2]. Male sterility encompasses genic male sterility (GMS), caused by nuclear genes, and cytoplasmic male sterility (CMS), caused by interactions between mitochondrial and nuclear genes [3]. Currently, CMS represents the most widely used breeding tool in cabbage hybrid seed production [4,5]. Ogura cytoplasmic male sterility (Ogura-CMS) was discovered in radish [6] and has been transferred to several *Brassica* species [5,7,8,9]. The original Ogura-CMS *B. oleracea* line exhibits poor agronomic traits, which have been improved by protoplast fusion [10]. Ogura-CMS is stable and easy to transfer between species; thus, it has become one of the most important types of CMS in *B. oleracea* [11].

In addition to the crucial breeding role of CMS in harnessing heterosis, it provides important materials for studying gametophyte development and mitochondrial–nuclear interactions, etc. [3]. CMS has been observed in approximately 200 species and is inherited maternally [12]. At least 17 CMS-related genes have been studied at the genetic and molecular levels [13]. These loci share similar characteristics, with CMS primarily caused by either novel mitochondrial open reading frames (ORFs), which generally result from rearrangements or recombination events in the mitochondrial genome [14]. Fertility restoration is mediated by nuclear-encoded fertility restorer (Rf) genes, most of which encode pentatricopeptide repeat (PPR) proteins that counteract the influence of CMS-associated genes [15,16].

Two hypothetical CMS pathways have been proposed: (I) CMS inhibits energy production by disrupting the mitochondrial electron transport chain complex; and (II) CMS impairs the normal growth of cells, at least in *E. coli* and/or yeast, because its products are cytotoxic [13,17]. In *Brassica* and *Raphanus* species, Ogura-CMS is caused by a mitochondrial gene named *orf138*, which encodes a subunit of a large mitochondrial membrane complex ORF138 protein [18,19,20]. Both plant membrane fractionation and analyses of *E. coli* have suggested that the ORF138 protein forms oligomers in the inner mitochondrial membrane of male-sterile plants, which is similar to another CMS protein, T-URF1321. Although the ORF138 protein severely inhibits bacterial growth, it does not affect respiration [21]. In a later study, Duroc et al. reported that the complex formed by the ORF138 protein in the inner mitochondrial membrane exerted an uncoupling effect because the mitochondria isolated from sterile plants consumed more oxygen than those of fertile plants, and this uncoupling effect was compensated at the cell and tissue levels, especially in vegetative tissues/organs, although the compensatory effects were apparently not efficient in male reproductive organs [17]. Despite these genetic and molecular studies, the mechanisms underlying the interference exerted by CMS genes on male gametophyte development are largely unknown.

High-throughput next-generation sequencing (NGS) has facilitated transcriptome analyses of Ogura-CMS materials (including Chinese cabbage, cabbage, broccoli) and may help provide a comprehensive understanding of the mechanisms underlying Ogura-CMS [22,23,24,25]. Xing et al. performed transcriptome and proteome analyses (focused on the transcriptome) and identified gibberellin, and sporopollenin synthesis as important pathways in Ogura-CMS cabbage [25]. Other studies performing proteomic analyses using two-dimensional gel electrophoresis (2-DE) have identified distinct differences in the proteomes of Ogura-CMS and fertile plants [26,27], including the down-accumulated protein species associated with processes that include carbohydrate and energy metabolism, cell wall remodelling, flavonoid synthesis and up-accumulated protein species like protease inhibitors.

Our group developed several elite Ogura-CMS cabbage lines with excellent agronomic performance [5], and they have been successfully used for the hybrid seed production of many elite varieties. F01-20 is an elite cabbage line originally introduced from Canada [28], and its Ogura-CMS line CMS01-20 was bred through crosses with a different Ogura-CMS line using F01-20 as the male parent and subsequent recurrent backcrossing with F01-20 for more than 20 generations. The very similar genetic backgrounds of this CMS line and its maintainer line make them ideal materials for cabbage breeding as well as for studying the molecular mechanisms of Ogura-CMS.

Herein, we describe the isobaric tags for the relative and absolute quantitation (iTRAQ)-based proteome analysis of Ogura-CMS using the cabbage lines CMS01-20 and F01-20. Our goals were to identify essential differential abundance protein species (DAPs) and pathways between male-sterile and male-fertile lines and investigate their potential mechanistic roles in Ogura-CMS.

## 2. Results

### 2.1. Morphological and Microscopic Examination

**Figure 1:**
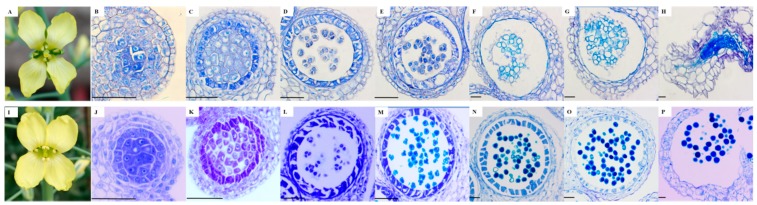
Phenotypes of Ogura cytoplasmic male sterility (Ogura-CMS) line CMS01-20 and its corresponding maintainer line F01-20. (**A**–**H**) CMS01-20; I-P: F01-20. (**A**) CMS01-20 shows degenerated anthers and no visible pollen; (**I**) F01-20 is fertility; (**B**,**J**) microsporocyte stage; (**C**,**K**) meiotic stage; (**D**,**L**) tetrad stage; (**E**,**M**) uninucleate stage; (**F**,**N**) bicellular microspore stage to trinucleate microspores stage. (**G**,**H**,**O**,**P**) mature pollen satge. Bar = 50 μm.

CMS01-20 showed degenerated anthers and no visible pollen compared with its maintainer line F01-20 (Figure 1A,I). We further observed male gametophytes of the two lines at different developmental stages using light microscopy (Figure 1B–H,J–P). No obvious phenotypic differences were observed before the tetrad stage. At the tetrad stage, certain tetrads exhibited irregular shapes. At the late tetrad stage or shortly after the release of microspores, CMS01-20 tapetal cells were swollen and vacuolated and the separation of microspores was delayed, indicating defects in the dissolution of the callose and tetrad walls. At the uninucleate to bicellular microspore stage, the tapetum layers of CMS01-20 showed earlier degradation, and we also observed the aggregation of abnormal and vacuolated immature pollen microspores as described in previous studies [23,29], although these microspores may be held together by the residue of degraded tapetum cells rather than by the tapetal layers. At the mature microspore stage, the aborted microspores were completely degenerated, no pollen was viable in mature locules, and the anthers did not dehisce.

### 2.2. Overview of the Protein Species Identified Using iTRAQ Data

**Figure 2:**
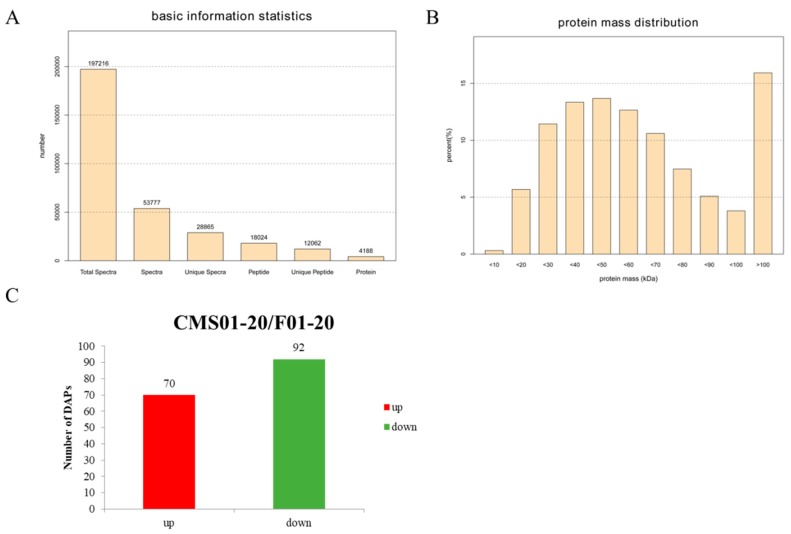
Protein species identification by the isobaric tags for the relative and absolute quantitation (iTRAQ) approach. (**A**) Number of spectra, peptide and protein; (**B**) percentage of protein mass distribution; (**C**) differential abundance protein species (DAPs) between CMS01-20 and F01-20.

iTRAQ-based proteomic analysis was employed to assess protein changes between the buds of F01-20 and CMS01-20. A total of 197,216 spectra were generated. After filtering the data with Mascot, 53,777 spectra were matched to known sequences, over half of which (28,865) were unique (Figure 2A). By searching against the cabbage A2 reference genome database, 12,062 unique peptides were identified within 4188 protein species (Figure 2A), which represented 11.8% of all predicted protein-coding loci in the genome. Most of the identified peptides had lengths between 8 and 16 amino acids. More than 60% of the protein species had masses between 30 and 70 kDa (Figure 2B), and approximately 70% of the protein species contained at least two mapped peptides.

To understand the functions of these 4188 protein species in cabbage buds, analyses were conducted with the Gene Ontology (GO), the Kyoto Encyclopaedia of Genes and Genomes (KEGG) and the Clusters of Orthologous Groups of proteins (COG) databases (Figure S1). The GO analysis showed that the biological process terms “metabolic process” (79.0%), “cellular process” (76.3%), “response to stimulus” (79.0%) and “single-organism process” (42.5%) were the most highly overrepresented functional groups; 10.8% of the protein species were involved in “reproductive process/reproduction”; the most overrepresented among the cellular component terms were “cell & cell part” (87.0%), “organelle” (72.6%), “organelle part” (38.6%) and “membrane” (34.9%); and the main for molecular function terms were “binding” (61.7%) and “catalytic activity” (57.4%).

### 2.3. Overview of the DAPs between CMS01-20 and F01-20

Following the criteria of a fold difference ≥1.2 and *p* value ≤ 0.05, we identified 162 DAPs between CMS01-20 and F01-20, of which 92 were down-accumulated and 70 were up-accumulated in the CMS line (Figure 2C, Table S1).

**Figure 3:**
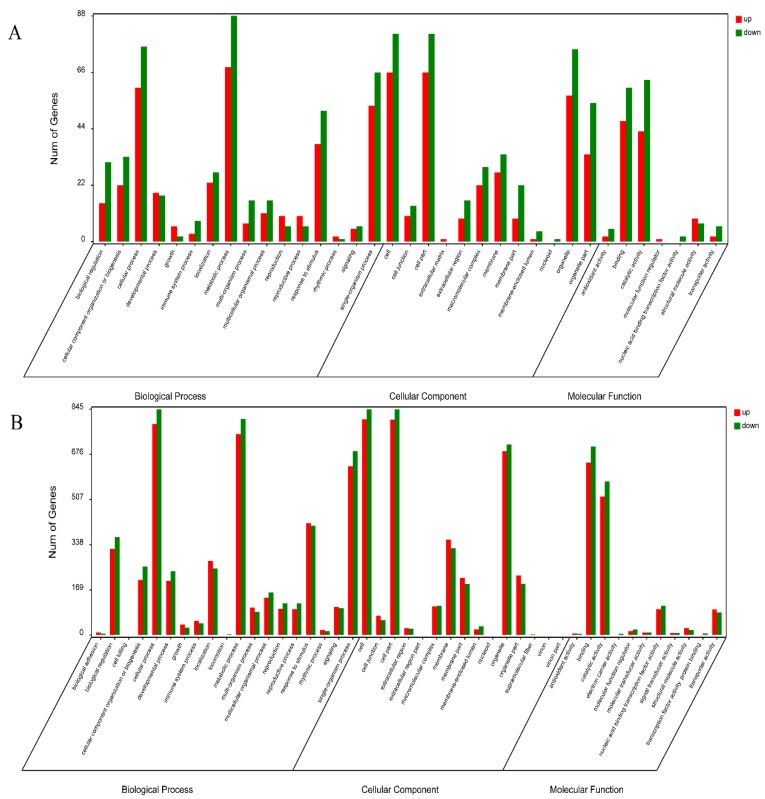
(**A**) Gene ontology categories for differential abundance protein species in the proteome data; (**B**) gene ontology categories for differentially expressed genes in the transcriptome data.

GO annotations were performed based on the TAIR GO Slim method provided by blast2GO. The GO annotations for 153 (94.4%) DAPs were divided into 35 functional groups, of which 16 were biological process GO terms (the largest category was “metabolic process”); cellular components accounted for 12 GO terms (the largest category was “cell”); and molecular functions accounted for 7 GO terms (the largest category was “catalytic activity”) (Figure 3A). Of the DAPs, 112 (70.4%) were assigned to 62 KEGG pathways, and enriched in 14 KEGG pathways (*p*-value < 0.05) including peroxisome (7.89%), cutin, suberine and wax biosynthesis (3.51%), sulphur metabolism (4.4%), ribosome (16.7%), tryptophan metabolism (5.26%), linoleic acid metabolism (1.75%), alpha-linolenic acid metabolism (3.51%), tropane, piperidine and pyridine alkaloid biosynthesis (3.51%), photosynthesis-antenna proteins (3.51%), carbon metabolism (14.9%), glyoxylate and dicarboxylate metabolism (6.1%), glutathione metabolism (5.3%), photosynthesis (5.3%), citrate cycle (the tricarboxylic acid) (4.4%) as listed in Table S2.

### 2.4. DAPs Involved in Oxidative Phosphorylation and TCA Cycle

Most CMS are associated with disturbances in the energy metabolism. The ORF138 protein formed a mitochondrial membrane complex that exhibited uncoupling effect, and affected oxygen consumption [17,21]. Thus oxidative phosphorylation and the TCA cycle are important pathways possibly affected by ORF138 protein. We found that all of the eight DAPs involved in oxidative phosphorylation were down-accumulated in CMS01-20 (Table 1), including one ETC complex I protein (Bol015119, NADH-ubiquinone oxidoreductase B18 subunit), one cytochrome *c* protein (Bol012326, cytochrome *c*), one complex IV protein (Bol010838, cytochrome *c* oxidase subunit Vc), and five complex V proteins (Bol009135 and Bol025034, ATP synthase subunit d; Bol017288, Bol025922 and Bol015469, ATP synthase 6 KD subunit). These results suggest CMS02-10 may have a decreased energy-generation capacity.

**Figure 4:**
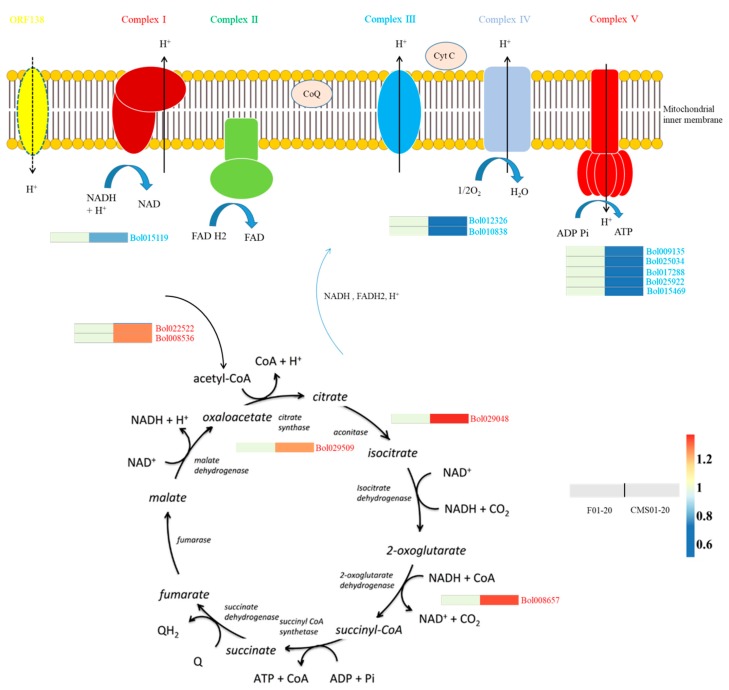
Differential abundance protein species involved in the oxidative phosphorylation and the tricarboxylic acid cycle. The possible uncoupling role of the ORF138 protein was also indicated on mitochondrial inner membrane. The fold changes of differential abundance protein species are indicated by the colour filled in the squares.

The citrate cycle (TCA cycle) provides NADH, FADH_2_ and H^+^ for oxidative phosphorylation. Five DAPs were annotated to be involved in TCA cycle: pyruvate dehydrogenase (Bol022522 and Bol008536) catalyses the synthesis of acetyl-CoA; citrate synthase 4 (Bol02950) catalyses the condensation of acetyl-CoA and oxaloacetate yielding citrate and CoA; aconitate hydratase (Bol029048) catalyses the conversion of citrate to isocitrate; 2-oxoglutarate dehydrogenase (Bol008657, 2-oxoglutarate dehydrogenase E1 component) catalyses the conversion of 2-oxoglutarate to succinyl-CoA. Interestingly, all the five DAPs were up-accumulated in CMS01-20, which may represent a compensatory mechanism triggered by the uncoupling effect of ORF138 protein [17], and this compensatory mechanism may be the reason for the increased oxygen consumption of male sterile plants [17]. The oxidative phosphorylation and TCA cycle network are shown in Figure 4.

### 2.5. Other Ogura-CMS Related DAPs and Pathways

**Figure 5:**
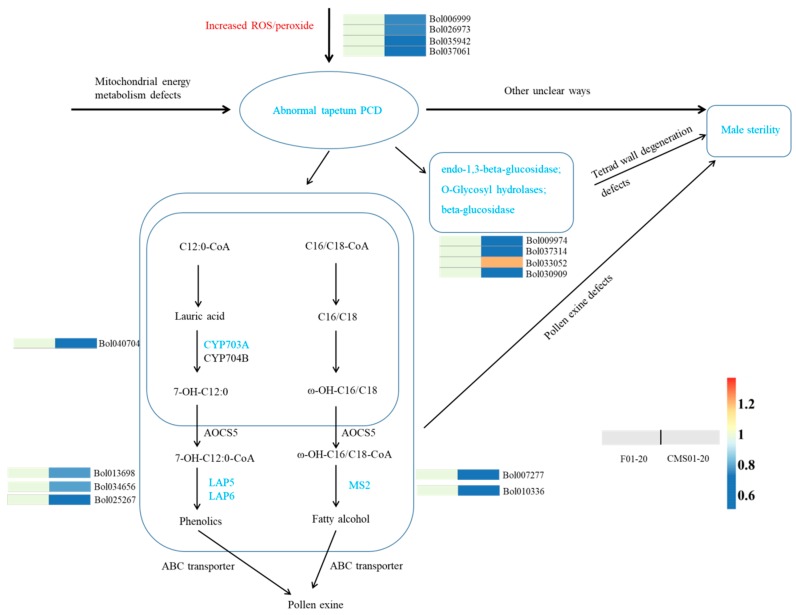
A possible network according to which abnormal tapetal programmed cell death is triggered by reactive oxygen species (ROS), resulting in male sterility. The differential abundance protein species involved in pollen exine formation and tetrad wall degeneration are also shown. The fold changes of differential abundance protein species are indicated by the colour filled in the squares.

Notably, terms related to cell wall assembly were significantly enriched among the DAPs. Fifty-five protein species were involved in cellular component organization or biogenesis, among which 33 were down-accumulated and 22 were up-accumulated in CMS01-20. We focused on protein species involved in the assembly (or degeneration) of pollen exine and the tetrad wall. We identified six DAPs involved in the synthesis of pollen exine. In the flavonoid biosynthesis pathway, LAP5 (Bol013698, Bol034656), LAP6 (Bol025267) and CYP703A/CYP703A2 (Bol040704) were down-accumulated in CMS01-20. In unsaturated fatty acid and fatty acid elongation pathways, MS2 (Bol010336, Bol007277) was down-accumulation in CMS01-20 (Table 1, Figure 5). These genes are vital for the development of viable pollen; therefore, they may be partially responsible for the phenotype of Ogura-CMS plants, such as the underdeveloped exine and the aberrant/aborted microspores observed here and in previous studies [23,29].

The degeneration of the callose wall and the outer wall (or pollen mother cell wall) is required for the release of microspores. Pectinase, endo-β-1,3-glucanases, exo-β-1,3-glucanase, and endo-β-1,4-glucanase enzymes are likely involved in this degeneration process [30]. We identified four DAPs that may be involved in the degradation of callose walls (Table 1, Figure 5). Bol009974 (predicted probable glucan endo-1,3-beta-glucosidase A6), Bol037314 (*O*-Glycosyl hydrolases family 17 protein) and Bol030909 (predicted beta-glucosidase) were down-accumulated in CMS01-20. Bol033052 (predicted beta-d-xylosidase, glycosyl hydrolase family 3) was up-accumulated in CMS01-20. They may be responsible for the pollen separation defects of CMS01-20 (shown in Figure 1). However, we did not identify any DAPs associated with pectin degradation.

Several previous studies have proposed a hypothetical mechanism in which CMS proteins trigger abnormal programmed cell death (PCD), which is usually associated with an increase in reactive oxygen species (ROS) and the release of cytochrome *c* in male organs, such as the tapetum [31,32,33]. Additionally, tapetal PCD often depends on the generation of ROS that can be detoxified by antioxidative enzymes including catalases [34,35]. In this study, we identified nine DAPs associated with PCD (Table 1, Figure 5). In the glyoxylate and dicarboxylate metabolism pathway, two catalase-3 proteins (Bol006999 and Bol026973) and one glycolate oxidase (Bol037061) were down-accumulated in CMS01-20. In the alpha-linolenic acid metabolism/linolenic acid metabolism pathway, four DAPs were down-accumulated in CMS01-20, among which allene oxide synthase (Bol035942) was a key enzyme catalysing the dehydration of the hydroperoxide to an unstable allene oxide. In the glutathione metabolism pathway, five down-accumulated DAPs and one up-accumulated DAP were identified. Among these five down-accumulated DAPs, Bol005496, Bol004624 and Bol033376 showed peroxidase activity. ROS may burst due to the down-accumulation of these enzymes and may trigger the abnormal PCD of the tapetum. Additionally, the disruption of allene oxide synthase DDE2 (the homologue of Bol035942) in *Arabidopsis* resulted in male sterility. Two oxygen-evolving enhancer protein species (Bol023353, Bol041074) were also associated with PCD and down-accumulated in CMS01-20, but they were predicted to be involved in the photosynthesis pathway and thus may not be CMS-related protein species.

We also identified DAPs and pathways that were similarly found in previous proteomic and/or transcriptomic analysis cases for male-sterile plants [25,36,37]. For example, the ribosome pathway (12 up-accumulated and 7 down-accumulated) and protein processing in the endoplasmic reticulum pathway (4 up-accumulated and 0 down-accumulated) was also identified in male-sterile cabbage and soybean [25,36,37], but their roles are largely unknown. For all the remaining DAPs and pathways, we did not find clues as to their possible roles in Ogura-CMS.

### 2.6. Joint Proteome–Transcriptome Analysis

To better understand the mechanisms underlying Ogura-CMS in cabbage, we analysed the transcriptomes of the CMS line CMS01-20 and its maintainer F01-20 via NGS. The RNA-seq libraries for F01-20 and CMS01-20 produced 91,952,648 and 98,470,304 clean reads, respectively. In total, 32,687 transcripts were identified for F01-20, and 32,680 transcripts for CMS01-20. A total of 3247 differentially expressed genes (DEGs) (*p* < 0.05) were identified, including 1525 up-accumulated and 1722 down-accumulated genes (Table S3). The GO analysis classified these DEGs into 45 GO categories, which showed a similar pattern to that of the DAPs (Figure 3B).

**Figure 6:**
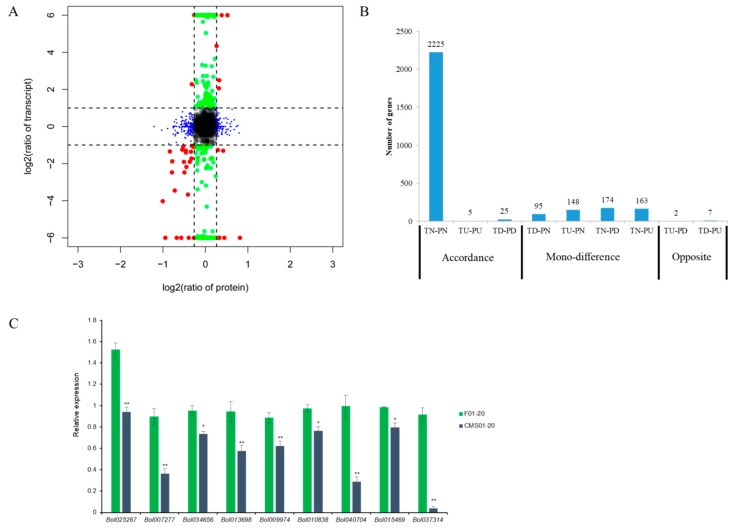
Integrative analyses comparing proteome and transcriptome data. (**A**) Genes were divided into nine groups according to log_2_ ratios of the protein species (*y*-axis) and transcripts (*x*-axis); (**B**) Number of genes among the nine groups in (**A**). T: transcript; P: protein species; N, no difference; U: up-accumulation; D, down-accumulation. (**C**) Expression validation for nine key genes by qRT-PCR. * *p* < 0.05, ** *p* < 0.01.

Integrative analyses comparing proteome and transcriptome data were performed between CMS01-20 and F01-20. The genes fell into nine groups based on the calculated log_2_ accumulation ratios of their protein species and transcripts (Figure 6A,B). These results showed poor correlation between the mRNA and protein species accumulation patterns as has been described in many previous studies [36,38,39]; this may be related to a combination of translational regulation, protein localization, protein modification, degradation, and other factors.

Although this conjoint analysis showed little overlap between the gene accumulation at the transcript and protein levels, certain noteworthy genes involved in sporopollenin synthesis (Bol013698, Bol025267, Bol034656, Bol040704, Bol007277), callose wall degeneration (Bol009974, Bol037314), and oxidative phosphorylation (Bol015469, Bol010838) showed accordance patterns. The expression levels of these nine genes were validated by qRT-PCR analysis, which revealed that all of the genes showed expression patterns consistent with the RNA-seq data (Figure 6C).

## 3. Materials and Methods

### 3.1. Plant Materials and Sample Preparation

Cabbage CMS line 01-20 (CMS01-20) and its maintainer line F01-20 were used in this study. These lines were sown on 20 August, transferred to a cold frame for vernalisation on 20 November and finally transplanted to a greenhouse on 3 March 2016 for bolting and flowering. All plant materials were obtained from the Institute of Flowers and Vegetables of the Chinese Academy of Agriculture Sciences (IVFCAAS, Beijing, China). During the flowering stage, flower buds with different lengths were sampled to observe a range of microspore developmental stages and identify differences between CMS01-20 and F01-20 using an Olympus CX31 optical microscope (Olympus Japan Co., Tokyo, Japan). Based on the microscopic examination results, flower buds before the bicellular microspore stage (≤3.5 mm) were collected for the transcriptome and proteome analyses. All collected buds were immediately frozen in liquid nitrogen and stored at −80 °C. Three biological replicate were performed for all experiments.

### 3.2. Microscopy

Flower buds with different lengths were fixed in formalin-aceto-alcohol (FAA), dehydrated in an ethanol series, embedded in paraffin, sectioned into 3–5 μm transverse slices using a microtome and stained with 1% toluidine blue as described by Lou et al. [40]. Then the anther transverse sections were observed with an Olympus CX31 optical microscope (Olympus Japan Co., Tokyo, Japan) and photographed with a Nikon 550D camera (Canon, Tokyo, Japan).

### 3.3. iTRAQ Analysis and Protein Species Annotation

The total protein species was extracted and subjected to iTRAQ labelling, strong cation exchange (SCX) separation and LC-electrospray ionization tandem mass spectrometry (LC-MS/MS) analysis using the same method as described by Chu et al. [41].

After converting them into MGF files, the raw iTRAQ data files were used for protein species identification and quantification. Database searches were performed using Mascot version 2.3.02 (Matrix Science, Boston, MA, USA) against a cabbage database, including 35,400 sequences from the *B. oleracea* genome A2 [42]. The search parameters were set as previously described [38].

Protein species with a fold change ≥1.2 (CMS01-20 vs. F01-20) and a false discovery rate (FDR) < 0.05 in at least two replicates were defined as differential abundance protein species (DAPs). All protein species identified were functionally annotated and classified based on Gene Ontology (GO) annotations (http://www.geneontology.org/), the Clusters of Orthologous Groups of proteins (COG) database (http://www.ncbi.nlm.nih.gov/COG/) and the Kyoto Encyclopaedia of Genes and Genomes (KEGG) database (http://www.genome.jp/kegg/pathway.html). DAPs were further analysed using the GO and KEGG databases to identify significantly enriched functional subcategories and metabolic pathways.

### 3.4. RNA-Seq Analysis and Conjoint Analysis with Proteome Data

Total RNA was extracted using an RNAprep pure Plant Kit (TIANGEN, Beijing, China) following the manufacturer’s instructions. High-quality RNA from each sample was used for cDNA library construction and RNA sequencing on an Illumina HiSeq 2500TM platform (Gene Denovo Biotechnology Co., Guangzhou, China). To obtain clean high-quality reads, adapter sequences, low-quality reads (>50% bases with Q-value ≤ 20) and unknown bases (>10% N bases) were removed from the raw reads. The short read alignment tool Bowtie2 [43] was used to map reads to a ribosomal RNA (rRNA) database. After removing the rRNA mapped reads, each sample read was then mapped to the reference genome (ftp://brassicadb.org/Brassica_oleracea/) with TopHat2 (version 2.0.3.12) [44]. Gene expression levels were normalized using the FPKM (fragments per kilobase of transcript per million mapped reads) method [45]. The edgeR package (http://www.rproject.org/) was used to identify differentially expressed genes (DEGs) between two samples. We defined genes with a fold change ≥2 and an FDR < 0.05 as significant DEGs. DEGs were then analysed for the enrichment of GO functions and KEGG pathways. GO terms or pathways with FDR ≤ 0.05 were defined as significantly enriched in DEGs.

For the conjoint analysis of DAPs and DEGs, the transcriptome and proteome data were combined using the same *B. oleracea* genome A2 database. Thresholds of “FDR ≤ 0.05, |log2FC| ≥ 1” and “*p* ≤ 0.05, |FC| ≥ 1.5” were set to select DEGs and DAPs, respectively. The correlation between the expression levels of the DAPs and their corresponding mRNAs were analysed by Pearson correlation tests.

### 3.5. Quantitative RT-PCR Analysis

Quantitative real-time RT-PCR (qRT-PCR) analyses were performed to validate the results from the DEGs. Total RNA was extracted from the buds of CMS01-20 and F01-20 plants using an RNAprep pure Plant Kit (TIANGEN, Beijing, China) according to the manufacturer’s instructions. RNA was treated with RNase-free DNase I (Fermentas, Harrington, QC, Canada) to remove genomic DNA. First-strand cDNA was synthesized using a PrimeScript 1st Strand cDNA Synthesis Kit (Takara, Kyoto, Japan). qRT-PCR reactions were conducted using SYBR Premix Ex Taq II (Tli RNase H Plus; Takara, Dalian, China) with a CFX96 Touch Real-Time PCR Detection System (Bio-Rad, Hercules, CA, USA). Three biological replicates (with three technical replicates for each biological replicate) were analysed for each gene. The relative expression level of each gene was estimated by the 2^−ΔΔCt^ method [46]. The *B. oleracea actin* gene (GenBank accession number AF044573.1) [23] was used as an internal control.

## 4. Discussion

As an important type of CMS in *Brassica* and *Raphanus* species, Ogura-CMS contributes significantly to hybrid seed production. Researchers have long been interested in Ogura-CMS, although the mechanisms underlying its ability to interfere with pollen development remain unclear. Previous transcriptome and proteome analyses of Ogura-CMS provided basic knowledge of the DAPs between Ogura-CMS plants and their maintainer lines [22,23,24,25,26,27]. iTRAQ is also an efficient and reliable approach for the relative and absolute quantification of protein species, and it has been applied in proteome analyses of CMS and GMS in several plants, including cabbage [25,37] soybean [36], cotton [38], and cybrid pummelo [47]. Herein, we reported the iTRAQ analysis of an elite cabbage Ogura-CMS line and its corresponding maintainer line. Many more protein species were identified using this method compared to the traditional 2-DE technique [26,27]. In total, 162 DAPs were identified, with 92 protein species down-accumulated and 70 up-accumulated in CMS01-20. These DAPs are mainly involved in carbon metabolism, energy metabolism, and cell wall assembly, etc.

Many CMS-related proteins are associated with deficiencies in the ETC and oxidative phosphorylation [3]. In sunflower, a chimeric mitochondrial ORF522 protein has been described that likely decreases ATP hydrolysis via mitochondrial ATP synthase [48]. In a CMS tobacco, the ATP/ADP ratio is significantly decreased in the floral buds of male-sterile plants [49]. In wild beet, mitochondrial gene *G* alters the molecular weight of a respiratory chain complex subunit, and the male-sterile *G* cytoplasm plants exhibit severely reduced cytochrome *c* oxidase activity [50]. In the HL CMS line of rice, ORFH79 disrupts the F_0_F_1_-ATPase, and reduced protein quantity and enzyme activity are observed in sterile plants [51]. In a more recent study, ORFH79 was confirmed to decrease the enzymatic activity of the mitochondrial ETC complex III by interacting with P61, a subunit of the ETC complex III, which resulted in a deficiency in ATP production and an increase in reactive oxygen species (ROS) content [52]. In the present study, we identified eight predicted ETC components differentially accumulated between the floral buds of CMS01-20 and F01-20. Five of these genes are predicted to be components of the mitochondrial ATP synthase complex and were down-accumulated in the CMS line, similar to a previous report in rapeseed [27]. Additionally, two cytochrome *c* (oxidase) genes and one NADH-ubiquinone oxidoreductase were significantly down-accumulated in sterile plants. These results suggest CMS02-10 may have a decreased respiration capacity, although Duroc et al. reported that ORF138 protein does not impair the capacities of electron transport chain complexes I, II, IV, or ATP synthase [17]. Interestingly, all DAPs involved in the TCA cycle were up-accumulated in CMS01-20. The TCA cycle provides precursors for many biochemical pathways and produces energy in the form of ATP largely via oxidative phosphorylation. The up-accumulation of TCA genes may compensate for the decreased respiration capacity and may be associated with the elevated O_2_ consumption rate observed in mitochondria from Ogura-CMS plants [17].

Pollen grains are covered by an exine wall that consists of sporopollenin, which provides essential protection from the environment and is involved in interactions with female stigma cells. Although the mechanisms of exine formation are not well understood, dozens of genes regulating sporopollenin biosynthesis have been characterized [53]. Six genes involved in sporopollenin synthesis were identified in the present study. Except for *Bol010336*, these genes were all down-accumulated at both the transcript and protein levels in CMS01-20, which was confirmed by the qRT-PCR analysis, whereas *Bol010336* was down-accumulated at only the protein level. *LAP5* (homologue to *Bol013698*, *Bol034656*) encodes an anther-specific chalcone and stilbene synthase (CHS) family protein, but does not present CHS activity in vitro, and it may act as a multifunctional enzyme or could be involved in a novel pathway for sporopollenin synthesis [54,55]. The mutation of this gene results in abnormal exine patterning. *LAP6* (homologue to *Bol025267*) is similar to *LAP5*, and double mutants of *LAP5* and *LAP6* exhibit strong male sterility because of a lack of exine on the surface of the pollen grains. *CYP703A/CYP703A2* (homologue to *Bol040704*) is specifically expressed in the anthers of land plants, and is involved in catalysing medium-chain saturated fatty acids and thus is essential for sporopollenin synthesis; moreover, *cyp703a* mutants produces pollen grains without exine, and they display a partial male-sterile phenotype [56]. *Male sterility 2* (homologue to *Bol010336*) is the first gene identified through a genetic approach using mutants with exine defects [57], and it encodes a fatty acid reductase that is responsible for the accumulation of C16 and C18 fatty alcohols, which are essential for pollen exine wall biosynthesis [58]. The homologue of this gene in moss shows a conserved function, suggesting that *MS2* is a core component of the sporopollenin biosynthetic pathway [59]. However, in Ogura-CMS plants, the down-accumulation of these protein species/genes may not explain the vacuolated and early degenerated tapetum phenotypes. Conversely, these genes may be down-accumulated because of the abnormal tapetum development (possibly caused by abnormal programmed cell death) because the tapetum supplies necessary metabolites, nutrients, and sporopollenin precursors for the normal development of the male gamete [60]. Indeed, Ogura-CMS tapetal cells showed reduced secretory activity [29]. Thus, other genes are likely involved in the impaired function of Ogura-CMS tapetal cells.

At the late tetrad stage, some key enzymes are secreted from tapetal cells to dissolve the callose wall and the outer wall. Three *quartet* (*QRT*) genes have been identified in *A. thaliana*, and they are involved in the pectin degradation of the pollen mother wall [61,62]. Mutants of these three *QRT* genes produce microspore tetrads that fail to separate, although these adhered microspores are viable [61,62]. Tratt reported other enzymes that are likely involved in dissolving the pollen tetrad walls, including endo-β-1,3-glucanases, exo-β-1,3-glucanase, and endo-β-1,4-glucanase [30]. In the present study, a light microscopy examination suggested a delayed separation of CMS01-20 microspores, which may be caused by these tetrad wall degradation-related genes. We also identified four down-accumulated genes that may be involved in the degradation of tetrad walls. In Ogura-CMS *Brassica napus*, Sheoran et al. also reported the down-accumulation of protein species associated with cell wall remodelling, including β-1,3-glucanase and pectinesterase using a 2-DE approach [27]. We also considered the down-regulation of these protein species/genes as a result of abnormal tapetal cells.

Premature PCD in the tapetum was observed in PET1-CMS cytoplasm sunflowers as indicated by cell condensation, oligonucleosomal cleavage of nuclear DNA, chromatin separation into delineated masses, and initial mitochondrial persistence [31]. This early PCD may be caused by the release of cytochrome *c* from the mitochondria into the cytosol of tapetal cells [31]. HL CMS rice showed a PCD phenotype in microspores accompanied by inner mitochondrial membrane disruption [32] that was triggered by chronic oxidative stress caused by increased ROS levels and reduced superoxide dismutase (SOD), ascorbate peroxidase (APX) and catalase activity in mitochondria. In wild abortive CMS (CMS-WA) rice, Luo et al. reported that WA352 interacts with nuclear cytochrome *c* oxidase 11 (COX11) to inhibit its function in peroxide metabolism, and this interaction was demonstrated to be responsible for premature PCD in the tapetum and male sterility [33]. Although PCD in the tapetum is a feature of normal development, premature or delayed tapetum PCD usually results in male sterility due to tapetum’s crucial role in pollen development [13,33]. Similar to several previous studies of Ogura-CMS plants, we observed a premature PCD phenotype in the tapetum [23,29], which may be responsible for the down-accumulation of many pollen wall assembly and pollen development-related genes, ultimately leading to male sterility. Therefore, identifying the key genes involved in tapetum PCD is crucial. In this study, we identified nine DAPs associated with PCD, especially, four protein species that are highly related to PCD: the two predicted catalase-3 proteins (Bol006999, and Bol026973, which catalyses the breakdown of hydrogen peroxide into water and oxygen), one allene oxide synthase (Bol035942, which catalyses dehydration of the hydroperoxide to an unstable allene oxide in the JA biosynthetic pathway), and one glycolate oxidase (Bol037061, which encodes a glycolate oxidase that modulates reactive oxygen species-mediated signal transduction). Decreased accumulation of these protein species, especially catalase and allene oxide synthase, may trigger PCD in the tapetum mediated by hydrogen peroxide or other ROS [13,63,64]. In addition, the homologue of Bol035942 in *Arabidopsis* is DDE2 (AT5G42650), which is an enzyme involved in jasmonic acid biosynthesis, and *dde2*-2 mutants show male sterility and exhibit filament elongation and defects in anther dehiscence [65]. Thus, *Bol037061* may be associated with the small indehiscent anther phenotype of Ogura-CMS.

## 5. Conclusions

The present study provided an iTRAQ-based proteome analysis of Ogura-CMS using the cabbage Ogura-CMS line CMS01-20 and its isogenic maintainer line F01-20. A total of 4188 proteins were identified, and 162 were designated as DAPs. Key pathways and DAPs involved in processes including energy metabolism in mitochondrion, assembly/degeneration of pollen exine and the tetrad wall, and programmed cell death were found to be closely related to male sterility. Transcriptome profiling revealed 3247 differentially expressed genes between the CMS line and the fertile line. Additionally, the integrative analyses of the transcriptome and proteome data revealed nine Ogura-CMS-related genes showing accordance accumulation patterns at the transcript and protein levels, and the expression levels of these nine genes were validated by qRT-PCR. This study improves our understanding of the genes associated with pollen development and the molecular mechanisms of Ogura-CMS.

## Figures and Tables

**Table 1:** Differential abundance protein species involved in oxidative phosphorylation, the tricarboxylic acid cycle, pollen wall, tetrad wall and programmed cell death.

	ID	Description	Up/Down in CMS Line
Oxidative phosphorylation	Bol015119	NADH-ubiquinone oxidoreductase B18 subunit	down
	Bol009135	ATP synthase subunit d, mitochondrial-like	down
	Bol025034	ATP synthase subunit d, mitochondrial-like	down
	Bol017288	mitochondrial ATP synthase 6 KD subunit	down
	Bol025922	mitochondrial ATP synthase 6 KD subunit	down
	Bol015469	ATP synthase 6 kDa subunit	down
	Bol012326	cytochrome c	down
	Bol010838	cytochrome c oxidase subunit Vc	down
TCA cycle	Bol022522	pyruvate dehydrogenase E1 component subunit beta-2	up
	Bol008536	pyruvate dehydrogenase E1 component subunit beta-2	up
	Bol008657	2-oxoglutarate dehydrogenase	up
	Bol029048	aconitate hydratase 1	up
	Bol029509	citrate synthase 4	up
pollen wall	Bol013698	LAP5; Chalcone and stilbene synthase family protein	down
	Bol025267	LAP6; Chalcone and stilbene synthase family protein	down
	Bol007277	MS2; fatty acyl-CoA reductase	down
	Bol034656	LAP5; Chalcone and stilbene synthase family protein	down
	Bol040704	cytochrome P450 703A2	down
	Bol010336	MS2; fatty acyl-CoA reductase	down
tetrad wall	Bol009974	probable glucan endo-1,3-beta-glucosidase A6	down
	Bol037314	*O*-Glycosyl hydrolases family 17 protein;	down
	Bol033052	beta-d-xylosidase 1	up
	Bol030909	beta-glucosidase 43 isoform X2	down
PCD	Bol006999	catalase-3	down
	Bol026973	catalase-3	down
	Bol035942	allene oxide synthase	down
	Bol037061	peroxisomal	down
	Bol005496	stromal ascorbate peroxidase	down
	Bol004624	glutathione S-transferase F9	down
	Bol033376	glutathione S-transferase F9	down

## Supplementary Materials

The following are available online at http://www.mdpi.com/1422-0067/19/10/3180/s1, Figure S1: Gene Ontology (GO) and Clusters of Orthologous Groups of proteins (COG) classification of total identified proteins. Table S1: Information of the DAPs between F01-20 and CMS01-20. Table S2: Kyoto Encyclopaedia of Genes and Genomes (KEGG) analysis of DAPs between F01-20 and CMS01-20. Table S3: Total number and bioinformatic analysis of differentially expressed genes (DEGs) between F01-20 and CMS01-20.
