# Supplementary figures and images for: iTRAQ-Based Proteomic Analysis of Ogura-CMS Cabbage and Its Maintainer Line
Source: Int J Mol Sci. 2018 Oct 15;19(10):3180. doi: 10.3390/ijms19103180 (PMC6214076; doi:10.3390/ijms19103180)

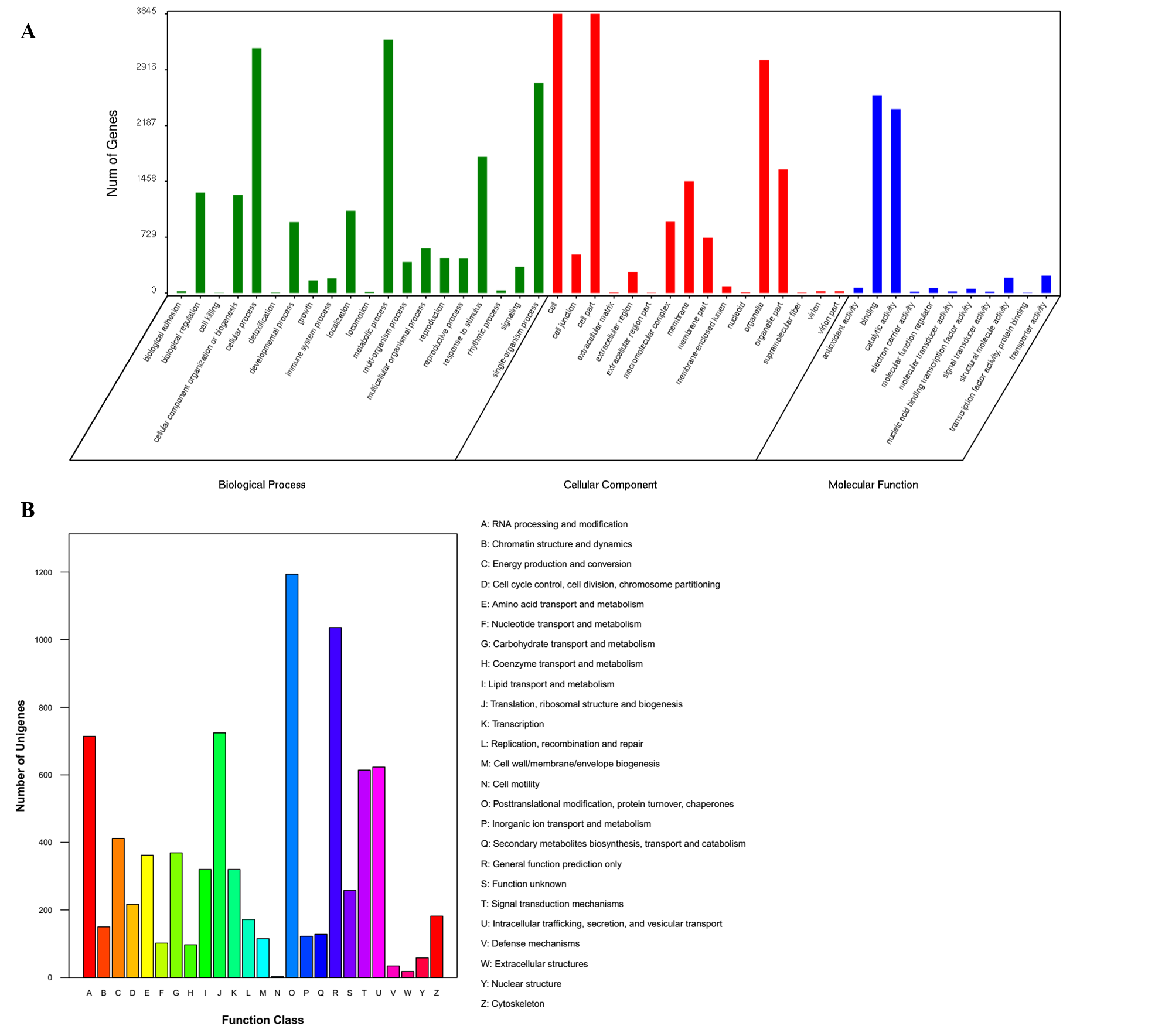

Supplement: Supplementary file 1 [file ijms-19-03180-s001.zip › Figure. S1.png]
